# Viewing Sexual Health Education through the Lens of Critical Pedagogy: A Case Study in Chicago Public Schools

**DOI:** 10.3390/ijerph18041443

**Published:** 2021-02-04

**Authors:** Elizabeth Jarpe-Ratner, Booker Marshall

**Affiliations:** 1 School of Public Health, University of Illinois at Chicago, Chicago, IL 60612, USA; 2 Office of Student Health and Wellness, Chicago Public Schools, 42 W. Madison St. Chicago, IL 60602, USA; elmarshall2@cps.edu

**Keywords:** comprehensive SHE, implementation of SHE, critical pedagogy

## Abstract

Comprehensive sexual health education (SHE) programs are being implemented in many state and local jurisdictions. Much research has focused on the strength and effectiveness of such programs. However, the experiences of teachers and students in their implementation is underexplored. A case study of the implementation of the SHE policy and curriculum in Chicago Public Schools sought to explore teachers’ and students’ experiences. Sixteen teachers were interviewed and five student focus groups, including 46 students, were conducted. Both teachers and students identified opportunities to improve upon the current program, including to (1) incorporate more student-centered learning opportunities and allow for tailoring to each specific group of students; (2) use discussion and dialogue to encourage students’ exploration of their own opinions and identities and development of a sense of agency over their own learning; (3) shift focus from risk reduction to a more holistic focus on healthy sexual wellbeing; and (4) directly discuss current health inequities, contributing factors, and intersectionality. These findings align with a critical pedagogical approach and underscore the need to understand SHE implementation within its sociopolitical context. Implications of the use of critical pedagogy as a framework for SHE in Chicago and beyond are discussed.

## 1. Introduction

Although tensions persist at the state and local level over whether and how to mandate sexual health education (SHE), many states, districts, and schools in the United States have adopted comprehensive SHE programs [1]. Comprehensive SHE curricula include information about building healthy relationships, including safer sex practices to reduce pregnancy and STI transmission, and focus on personal skill development including communication and decision making [2,3]. Many such programs are also intended to be LGBTQ+ inclusive [1,2,3]. Comprehensive SHE programs are being adopted in response to strong evidence of their effectiveness [2] as well as in light of persistent health inequities, particularly in communities of color, and particularly among students identifying as LGBTQ+ [4,5,6]. Chicago Public Schools’ (CPS) 2013 policy and accompanying curriculum is one such example [7]. The curriculum is aligned with the National Sexuality Education Standards [8] and is designed to be developmentally appropriate, medically accurate, and LGBTQ+ inclusive. In places such as Chicago, where comprehensive SHE has been adopted, focus must shift to its implementation.

Noted challenges in implementation across schools nationally include a lack of funding and materials, staff training, and perceived or actual parent pushback [9,10]. In Chicago specifically, additional challenges include a lack of planning time and dedicated time in the school schedule [11]. These challenges underscore the need to better understand teachers’ own experiences implementing the curriculum. Implementation studies of school health programs more generally point to teachers’ positive experiences being associated with higher levels of implementation (e.g., higher numbers of lessons and proportion of lesson content reported as implemented) [12,13]. Further, positive student experiences are associated with student engagement, which is essential to learning outcomes across all curricular areas [14,15], and SHE should be no exception. This study aimed to explore the current state of SHE curriculum implementation from the perspective of both teachers and students.

The primary goals of CPS’s policy, and others like it, are to address health inequities and ensure LGBTQ+ inclusivity. Students living in Chicago’s most disinvested communities, particularly students of color as well as those who identify as LGBTQ+, disproportionately experience persistent rates of STI transmission [16,17], sexual risk-taking behaviors, and mental health problems [18,19]. These health inequities result from centuries of structural and institutional racism and discrimination [20,21]. Educators outside the field of SHE have sought to acknowledge such structural and systemic inequities directly in their teaching through the lens of critical pedagogy. Critical pedagogical practices prioritize the voices and lived experiences of the students in their classrooms and actively push against the tendency to discount their lives and experiences as so often happens in other societal domains [22]. Further, such practices facilitate student development of a sense of agency for altering inequitable conditions [23,24]. This study sought to explore whether and how the frame of critical pedagogy fits with the ways in which students and teachers are experiencing SHE and their recommendations for improvements.

This study aimed to answer the following research questions: (1) What are the current experiences of teachers and students of the CPS curriculum? (2) What is recommended to improve upon those experiences? and (3) What role might critical pedagogy play in either current implementation or in recommendations for curricular changes? The central proposition of this study is that a curriculum that is more easily implementable by teachers and more engaging to students will better facilitate student learning, thereby contributing to elimination of sexual health inequities in Chicago.

## 2. Materials and Methods

This case study of CPS’s implementation of its own mandated SHE curriculum was developed in consultation with CPS stakeholders from the Office of Student Health and Wellness (OSHW) who oversee the policy’s implementation. Although the policy and curriculum require K-12 instruction, this study focused on high schools. At the time of this study’s conceptualization, during the 2015–2016 academic year, among the approximately 300 CPS schools where SHE was implemented at all, implementation rates were known by OSHW to be highest among 9th-grade teachers [11]. This is due in part to the fact that there is a graduation requirement that high school students take a health education class in the 9th grade.

An initial list of 67 teachers who had implemented SHE with 9th graders during the 2015–2016 academic year was generated based on OSHW’s implementation data. All teachers were asked via email if they were still teaching 9th-grade SHE. Of the 47 that responded, 29 were still teaching 9th-grade SHE. Targeted email invitations were sent as follow up by the lead researcher using a purposeful sampling approach [25] to ensure that the schools represented in this study included a mix of the city’s geographic regions, types of high schools, and predominant race/ethnicity of students. Examples of types of high schools in CPS include neighborhood schools (where enrollment is based on living in proximity to the school) and selective enrollment (schools that enroll using a merit-based application process). Four teachers declined enrollment. Sampling was further guided during data collection through the identification of common themes to ensure that saturation [26] was achieved for both the focus groups and interviews.

Data were collected during the 2016–2017 academic year. All study procedures were approved by the University of Illinois at Chicago Institutional Review Board (protocol #2016-0010) and Chicago Public Schools Research Review Board (protocol #1143). Teachers provided written consent prior to participation and received twenty-dollar gift cards as an incentive. Students’ parents/guardians signed a parent permission form and students provided written assent prior to participation. Students received ten-dollar gift cards as an incentive and were provided with a complimentary lunch.

A total of three researchers (the lead researcher and two research assistants) were involved in collecting and analyzing these data. At the time, the lead researcher had nearly a decade of qualitative research experience and five years’ experience conducting research in school settings. Their roles are described in greater detail below.

### 2.1. Data Collection

#### 2.1.1. Interviews

Of the 67 SHE teachers who were initially contacted, 16 were still teaching 9th-grade SHE, were interested in participating, and represented a mix of schools, as described above. During semi-structured interviews conducted by the lead researcher, teachers were asked to reflect on their own implementation experiences utilizing the CPS curriculum. The semi-structured interview guide included questions about what content they implemented, what may have been left out, what they thought worked well, what could have worked better, what they may have changed, and why. Probes related to reasons for curriculum adaptations, drawn from the literature, such as familiarity with the content, comfort with the content, perceptions of student engagement with the content, were asked to assess reasons for reported changes or revisions to the curriculum. Additionally, teachers were asked about their perceptions of student engagement and student learning. Again, probes pulled from the literature, such as the use of student-centered teaching strategies, were used to more fully assess perceptions of student learning. A CPS SHE teacher (who had since moved to a different position and was not eligible at the time of this study) reviewed the semi-structured guide to assess interpretability, sequencing, and language. Before the interview, teachers were asked to completed a short demographic questionnaire indicating age, race/ethnicity, and gender identity, and type of teaching position that they hold (e.g., PE/Health teacher and Special Education). Interviews ranged from 30 to 60 min; most were conducted in person and a few were conducted via phone. All interviews were recorded and transcribed.

#### 2.1.2. Focus Groups

The focus group topic guide used with all groups had questions that explored students’ own perspectives on how engaged they were in the curriculum, what content they recalled, what content may have been missing from their perspective, what teaching strategies were used that facilitated learning, and what could have been improved about the content or delivery from their perspective. Two recruitment strategies were used to assemble student focus groups. Two of the groups were recruited in collaboration with teachers who had participated in this study. Teachers distributed parent/caregiver permission forms and arranged time and space for the groups. Three groups were recruited through their school Genders and Sexualities Alliances (GSAs), clubs led by LGBTQ+-identified students and their allies. Adult sponsors of the groups assisted in distribution of parent/caregiver permission forms and arranged time and space for the groups. All groups lasted approximately 60 min and were audio recorded. At the beginning of the group, students completed a short demographic form indicating age, race/ethnicity, and gender identity. The lead researcher acted as facilitator for all groups; one research assistant served as note taker for two groups and another research assistant served as note taker for the other three groups. All recordings were transcribed following the groups.

### 2.2. Analytic Approach

#### 2.2.1. Data Analysis

Interview and focus group data were analyzed using the constant comparative method [27]. The lead researcher initially reviewed all transcripts to assess relevance of a priori codes and to begin to identify emergent codes. Examples of a priori codes, drawn from the health education implementation literature, included “curriculum adaptations” with associated subcodes such as “lack of comfort,” “need for more student-centered approach,” and “need to change to strategy to match student learning modality.” Emergent codes included “responding to students’ stated interests” and “need for updated material.” “Recommendations” for changes to the curriculum content and delivery were coded for teachers and students. Specific code families were designated for application to either focus group data, interview data, or both.

The lead researcher and the research assistant who had served as note taker for three of the focus groups each coded a subset of transcripts (4 teacher interview transcripts and 3 student focus group transcripts). After comparing coded text segments, they discussed areas of disagreement and made revisions to the codebook to attempt to increase clarity and consistent application of codes. After two rounds of coding, the same transcripts, an 80% agreement rate was reached on both the focus group and the interview transcripts. Dedoose software was utilized during this analysis [28]. After undergoing this round of first cycle coding [29], the lead researcher identified key themes related to the first two research questions: (1) What are the current experiences of teachers and students of the CPS curriculum? and (2) What is recommended to improve upon those experiences? A data display matrix [29] was used to facilitate comparisons between teacher recommendations and student recommendations.

As the recommendations were identified through the first cycle coding process described above, the lead researcher began to recognize distinct patterns and themes. These included the following: (1) instructor training recommendations that were shared with OSHW directly, (2) LGBTQ+ inclusive SHE recommendations which have been reported on elsewhere [30]; and (3) elements of critical pedagogy. A priori codes were then developed based on the critical pedagogy literature and these codes were applied through second cycle coding [29] to the quotations coded as “recommendations” in first cycle coding. Themes were developed from this coded data to answer the third research question: What role does or could critical pedagogy play in either current implementation or in recommendations for curricular changes? Again, a data display matrix [29] assisted in this comparison.

#### 2.2.2. Member Checking

The lead researcher engaged in ongoing dialogue with OSHW team members at CPS as well as key student stakeholders working on student leadership teams at community-based organizations, and key community-based stakeholders, including community-based sexual health educators, throughout the process. This involved informing the design and instrument development as well as interpretation. The sharing of initial findings was performed as a form of member checking in order to ensure trustworthiness of this study’s findings [31] as well as to ensure that this study’s recommendations were interpretable and useful to key stakeholders.

## 3. Results

Findings are presented in three subsections. The first describes the demographic characteristics of the teachers and students in this study. The second details key themes that emerged from this study related to the question of teacher and student experiences of the curriculum’s implementation and opportunities to improve upon those experiences in order to better facilitate student learning. Finally, a third subsection outlines the ways in which these findings align with key characteristics of critical pedagogy. 

### 3.1. Sample Characteristics

There are sixteen schools represented in this study. Two schools had both a teacher participant and also served as the basis for student focus groups. Another school had two teacher participants. The types of schools represented in this study are listed in Table 1. Most were neighborhood and selective enrollment schools (neighborhood schools are open to all students living within the school’s boundaries and selective enrollment schools accept students through a competitive application process). There was a relatively even mix of schools in which the predominant student demographic group was either African American/Black or Hispanic/Latinx, or a mix of demographic groups. Selective enrollment schools and schools with a mix of demographic groups are over-represented in this sample as compared to the overall distribution of such schools across the district [29].

A total of 16 teachers participated in this study. Demographic characteristics of teacher participants are shown in Table 2. Most teachers identified as White and as PE/Health teachers; half identified as male and half as female.

A total of 46 students participated in this study. Table 2 also depicts demographic characteristics of student participants (note that this same dataset and sample characteristics have been reported on elsewhere albeit related to a distinct set of research questions [30]). Most students identified as female or girls; most identified as students of color, with two White students participating. Although the types of schools did not reflect the distribution across the district, the race/ethnicity of students in this sample does more accurately reflect the proportions at the district level [32].

### 3.2. Key Themes

Upon analysis of the interview and focus group data, four themes, or recommendations, emerged describing what teachers were doing to enhance the curriculum and what students indicated was either valuable or in need of improvement. These include the following and are described in detail below: (1) incorporate more student-centered learning opportunities and allow for tailoring to each specific group of students; (2) use discussion and dialogue to encourage students’ exploration of their own opinions and identities and development of a sense of agency over their own learning; (3) shift focus from risk reduction to a more holistic focus on healthy sexual wellbeing; and (4) directly discuss current health inequities, contributing factors, and intersectionality.

#### 3.2.1. Incorporate More Student-Centered Learning Opportunities and Allow for Tailoring to Each Specific Group of Students

A key finding that emerged both from teachers and students was the impression that the curriculum was not sufficiently engaging to students; too many of the lessons within the curriculum relied on lectures and slide presentations as primary instructional strategies. This was perceived by both teachers and students as not conducive to student engagement. Many of these lessons were noted as “dry” and “boring.” As one teacher noted:

*Those Power Points were awful and kids got bored with them… [one lesson] was going over like the laws and stuff, and my kids did have a lot of questions about it… but the writing [the ways it was presented in the slides] was just way too much for kids to fully take in.*

Teacher #2

Other teachers noted that information was out of date on some of the slides. For example, risk behavior statistics were several years out of date and this had turned students off.

Not only were slides noted as boring or out of date, teachers also noted that is no longer considered a “best practice” for teaching.

*If you look around, not many teachers are doing straight lecturing any more. That’s really not a practice that we’re following these days. We want to make sure that the student has more control. Discovery I think is important, so having them discover the answer as opposed to me just delivering the answer.*

Teacher #12

Teachers specifically mentioned that the curriculum should be more “student centered” and should offer more opportunities for “hands-on” learning that engages their students. Teachers discussed making changes such as creating team-based games, bringing in more videos, offering more opportunities for individual and group projects, and the opportunity for students to conduct their own research projects.

Further, teachers articulated a need for tailoring teaching strategies to the students in each class and each year. They requested that the lessons include more suggestions for adaptations or differentiation of content for different levels of prior knowledge, different classroom dynamics, and students’ learning styles. As the teacher whose quote is noted in Table 3 explained, some groups are ready to engage in deeper conversation with one another based on prior knowledge and/or trust and comfort with one another, while other classes are not. Each require different approaches and the curriculum should contain approaches that could be used in these different scenarios. One teacher pointed out that allowing for this tailoring is part of making the curriculum truly “student centered.”

Students agreed that there is a need for more student-centered engagement and opportunities for experiential learning. They noted that they preferred projects and games and also noted that they appreciated when teachers took suggestions from them about what works best and incorporated their feedback into future lessons. Further, they specifically stated that there is an opportunity for discussion and dialogue which brings us to the next recommendation.

#### 3.2.2. Use Discussion and Dialogue to Encourage Students’ Exploration of Their Own Opinions and Identities and Development of a Sense of Agency over Their Own Learning

As noted above, teachers and students discussed the need for more experiential learning activities, such as games and projects. However, another teaching strategy that teachers had employed and that students had experienced was group dialogue and discussion. Both teachers and students stated that that curriculum should allow for more opportunities for discussion among students and between teachers and students. Approximately one-third of the interviewed teachers discussed employing this strategy and adapting existing lessons to increase the opportunity for students to engage in dialogue. This was noted as being performed as a class so as to allow the teacher to facilitate the dialogue as well as in small groups and pairs to allow all students to engage in discussion with one another.

Teachers noted that adding in opportunities for discussion was one of the adaptations that they often make with the curriculum. However, as this teacher notes below, this sometimes involved a greater level of flexibility in allowing activities to take longer than planned.

*So I go by how much the kids get out of it. If it’s going to take more than a class period, because we got a lot of good class discussion going on I’m not going to stop it and move on… The kids, if they have questions and there’s good group discussion I’m not stopping.*

Teacher #8

Students agreed that time for discussion is crucial in SHE classrooms. Some had experienced teachers allowing time for discussion and valued this approach. As this student noted, part of the value of this approach is fostering dialogue among students and not just between students and the teacher.

*So when you go ahead and pull out whoever has a question [from the question box], SHE also not only gives her answer to it, but SHE lets the other students if they have an answer as well, and she’ll agree or disagree and explain what they’re really saying, if no one else really understands what the student is saying. I feel like that is really good, because it’s not just her answering the question, also being engaged with the class as well.*

Focus group #3

Other students spoke about how they would recommend such an approach as part of an ideal SHE classroom. Again, like the first recommendation noted above, this student contrasted the discussion-oriented approach with the lecture-based approach most students and teachers had noted as a drawback of the way the curriculum had been structured.

*[If I were a sex ed teacher] it’s just more of a discussion thing more than me standing up in front of the board and teaching you from a power point, or from writing on the white board… so like everyday I would probably allow my class to have [a discussion], because I think that that’s creative and think that’s a good way to still keep order in the class but allow them to share their opinions…*

Focus group #2

Students recommended this approach, not only because it was considered more engaging, but also because of the ways that it can help underscore the message that students are responsible for making their own informed decisions. Students spoke emphatically about the ways in which discussion-oriented approaches can foster students’ own development of agency over their decisions.

*Nothing was really forced on us, as to how to think, what to do, all of that. It was like I’ve been repeating is that the decision is up to us. I think she probably was stressing on that the entire time. Whatever we do, the choice is up to us.*

Focus group #3

Teachers acknowledged that approaches through student dialogue with one another engender more accountability or responsibility for their own learning. As this teacher pointed out, centering students in the classroom keeps them engaged, and helps them take ownership over their own learning.

*I think it’s important to always focus on how are we getting students to learn from one another? How are we getting kids up out of their seats and learning on their feet? Like I said, the importance of prenatal care, where you have kids moving around or working together to try to figure out these definitions. Just seeing where you have kids up and engaged is always going to help… It gives the kids some responsibility to learn on their own as well.*

Teacher #4

#### 3.2.3. Shift Focus from Risk Reduction to a More Holistic Focus on Healthy Sexual Wellbeing

While there was strong agreement between teachers and students about the need to move towards more student-centered teaching practices and allow opportunity for student discussion, there was more divergence regarding specific content recommendations.

Students were emphatic that the content of the current curriculum was too narrowly-focused on risk and risk-reduction.

*When we talked about it, it was always male and females. Nothing else was ever brought up about other situations. It was always, “Don’t get pregnant. Don’t ruin your future.”*

Focus group #2

As noted in Table 3, students stated that not only is risk overemphasized but there was no acknowledgement that sex is for pleasure; the only purpose noted was reproduction. Students in one of the GSA-recruited focus groups specifically noted that leaving pleasure out of the SHE classroom is antithetical to the idea that the curriculum is intended to be LGBTQ+ inclusive.

Further, students noted that rather than focus on covering every purpose or form of sex and sexual behavior, the prevailing message coming from the curriculum should be normalization and affirmation of a range and diversity of sexual behaviors and relationship structures. They stated specifically that the message should be that all forms of healthy relationships, all forms of sexual behavior using safer practices, and all identities are normal. They stated this was important so as to avoid stigmatizing specific identities, behaviors, and types of relationships.

Teachers did acknowledge the need for the inclusion of a broader content base and the need to define sexual health in a more holistic way that incorporates mental and emotional health. They expressed a need for greater emphasis on healthy relationships. Some also noted a need to specifically focus on mental health and the connections between healthy relationships, self-esteem, body image, and emotional health. Several teachers discussed the need for the curriculum to be more inclusive of LGBTQ+ relationships. Many noted that LGBTQ+ content, mental health content, and trauma are all areas in which they need more training and resources. Notably, no teachers in this sample discussed a need to include sexual pleasure as a topic.

#### 3.2.4. Directly Discuss Current Health Inequities, Contributing Factors, and Intersectionality

Like the previous recommendation, students were much more adamant than their teachers about the need to explicitly address health inequities in the SHE classroom. Notably, two of the three teachers of color in this sample discussed ways in which they had implemented the curriculum in a way that explicitly addressed health inequities. None of the White teachers expressed having done so. A teacher who identified as a Black female talked about using songs and media images to deconstruct messaging, specifically focused on the sexualization of women and girls of color. Another teacher who identified as a Black male noted the importance of discussing how certain communities had experienced discrimination and institutional racism contributing to current health inequities.

A larger number of teachers noted the importance of highlighting stories from the students’ own communities. One teacher brings back a former student who is living with HIV as a guest speaker to share their story of contracting and living with the virus. Another teacher brought in a local news story about a teen who had experienced sexual assault as an entrée to discussing sexual assault, trauma, mental health, and coping strategies.

Teachers expressed their belief in the importance of connecting to students’ lives and communities. However, with the exception of the two teachers of color noted above, they seemed to fall short of connecting these experiences to systems of oppression and structural and societal forces that lead to existing inequities experienced by people of color. Most teachers in this study noted their discomfort in talking about race, ethnicity, and inequities; for example, they were hesitant to discuss risks associated with specific demographic groups. They expressed concern about not wanting to further stigmatize these groups and/or not knowing how to discuss increased risk without “blaming the victims.”

*There’s always going to be a risk–guy to guy–girl to girl–I don’t like getting into this “has a higher risk than”–no one is exempt–there is no disease that discriminates against race, gender, etc.*

Teacher, #16

When asked about training needs and perception of needed skills development, nearly all teachers in this sample stated that they needed more support in addressing LGBTQ+ topics. Three teachers stated that they needed help in addressing structural racism, societal messages directed towards people of color, and/or health inequities.

In contrast, students were particularly vocal about the need to address both the inequities faced by youth of color as well as youth identifying as LGBTQ+ and there is a need to contest myths, stereotypes, and discrimination explicitly. As the quotation from the student in Table 3 notes, this was not something that many students had yet experienced in their SHE classes and this recommendation reflects what many saw as an essential ingredient to ideal SHE. Students also noted a need to bring identities into the classroom and acknowledge the ways in which identities intersect and how that may influence sexuality.

Students identifying as straight and/or cisgender stated that they needed to discuss these topics related to LGBTQ+ identities explicitly so as to learn how and when to use language and discuss identities in respectful and affirming ways. Some students likened this to the ways in which men need to learn how to discuss sexism and how Whites and non-Black people of color need to learn how to discuss anti-Black racism in respectful and appropriate ways.

In their own ways, both teachers and students expressed a lack of comfort in discussing these topics. Importantly, students are recommending these be brought directly into the SHE classroom so they can learn how to move past this discomfort.

### 3.3. Alignment to Key Dimensions of Critical Pedagogy

The recommendations made by teachers and students in this study align with central tenets of critical pedagogy, as outlined in the third column of Table 3. The first recommendation, for more student-centered learning, aligns with the idea espoused by critical pedagogy scholars that by using lecture-based approaches that are teacher centered, rather than student centered, teachers run the risk of reverting to assumptions that students passively take in knowledge [30]. Students and teachers in this study were clear that to foster engagement and learning, activities must be student centered and that sometimes this requires tailoring teaching strategies to the needs of specific students or classes, and this idea that teaching should connect to students’ own lived experiences, assets, and needs is also central to critical pedagogy [20].

The second recommendation called for more discussion and dialogue. Specifically, students asked for more opportunity to discuss and explore identity. Additionally, students and teachers acknowledged that through discussion and dialogue, they might assert a sense of agency over their own learning; this too is a central tenet of critical pedagogy [21,22].

The third recommendation called for a more holistic approach to exploring sexuality and sexual health topics that is not so centered on what not to do, or on how not to get pregnant. This aligns with the critical pedagogical approach of getting away from focusing on deficits, needs, and risks, to instead focus on assets, resources, and resilience [31]. This aligns with the idea that students and teachers discussed a need to focus on the active development and attainment of sexual health that encompasses mental, emotional, and physical health, rather than simply a focus on risk avoidance.

Finally, the fourth recommendation is to more explicitly include a discussion of inequities and the history and current conditions that result in current inequities. This is perhaps most directly aligned with critical pedagogy’s core tenet of using the classroom as a venue to teach about systems of oppression as well as encourage the critique of those systems [21,22]. This aligns with the idea that classrooms provide opportunities and space for not only actively pushing against the tendency to discount the lives of students of color and LGBTQ+ students as so often happens in other societal domains, but to center those lives and lived experiences as central to learning, exploration, and identity development [20,32].

## 4. Discussion

This study reveals how teachers and students are experiencing an evidence-informed SHE curriculum. This study investigates how teachers are implementing the curriculum, what changes they are making, and what they recommend to improve it. This study also explores how students are experiencing the curriculum and what recommendations they have for changes that they would like to see. Students and teachers report a desire for more student-centered, engaging lessons as well as more time for discussion and dialogue. Students request a more positive, asset-based approach as well as more explicit discussion of inequity, identity exploration, and acknowledgement of intersectional identities and the role they play in sexuality. As described above, these four recommendations align with critical pedagogy concepts. As this was an evaluation conducted in collaboration with CPS, first we discuss local next steps and implications and then we turn to larger implications within the field of SHE.

The results of this study were first shared with the OSHW during the 2017–2018 school year. The district released a revision of the curriculum in school year 2019–2020. In this revision, much of the teaching strategies have been adjusted to be more student centered and there is more content related to identity, particularly LGBTQ+ identities. For example, high school curricula now include a structured debate activity as well as student advocacy presentations focused on the healthcare rights of adolescents [36]. Additionally, as of December 2020, the district passed an updated version of its SHE policy, which now requires that the SHE programming: “is culturally, developmentally, linguistically, and age appropriate; provides strategies to support all students that are inclusive of gender identity, gender expression, sexual orientation, sexual behavior, race, and disability; and is guided by anti-racist pedagogy” [37]. Finally, as of the 2020–2021 school year, the district began requiring all staff to complete an OSHW-created virtual training, “Supporting Transgender, Nonbinary and Gender Nonconforming Students.” The training takes an intersectional lens to discuss the structural oppression which leads to health inequities among students. The updated curriculum, the new policy, and this new training are currently undergoing evaluation and will be reported on in the future.

In turning to this study’s implications for the sexual health field more generally, this study provides empirical evidence supporting the use of critical pedagogy as a framework for addressing teacher and student SHE desires and needs. The use of such a framework has the potential to address noted gaps in SHE research, heed calls for improvements in SHE made by advocates, young people, and educators, and underscores existing SHE efforts led by BIPOC and LGBTQ+ educators.

First, there is a persistent gap in SHE research related to the recommendations made by teachers and students in this study. It has been noted that most of the research in SHE centers around two areas: (1) intervention research focused on understanding the impact of SHE on health outcomes; and (2) critique of SHE curricula from a feminist, queer, and critical race theory lens [38]. There has been a relative dearth of research focused on practice, teaching strategies, and student engagement [38,39]. Issues of teaching strategy and student engagement were central to the teachers and students in this study and these topics deserve greater research attention. The use of a critical pedagogical framework may be an avenue to address this gap while also bridging existing bodies of research, including previous SHE calls for inclusion of pleasure [40,41] and a broader range of sexual health topics that extend beyond a focus on reducing risk [42]. While the potential value of critical pedagogy in SHE has not been widely discussed, it has been mentioned by some [4,43] and this study further supports this call. Furthermore, the handful of SHE studies that have incorporated student experiences, align with findings reported here, including the need for content that is more relevant to students’ own lives, the elimination of the stigmatization of LGBTQ+ identities, and the use of strategies that welcome questions and promote dialogue [44]. The findings here strongly support the need for more LGBTQ+-inclusive SHE that has been called for in these other studies. Findings from this same dataset related to LGBTQ+ inclusivity specifically are described in another article [30].

Although teaching strategies have not been widely discussed in the SHE literature, there has been an emphasis in the health education literature on the need to minimize curriculum adaptation and maintain fidelity to curricula [45,46]. This has resulted in tension in among SHE curriculum developers, researchers, and instructors. Instructors are told that they must adhere strictly to program content in order to ensure positive health outcomes are achieved, resulting in their practice-based evidence is ignored. While this is explored in greater depth in another article from this study [47], it is important to point out here that tailoring and adaptation to a particular student or class is perceived by teachers and students to increase engagement. Therefore, some amount of tailoring and guidance around adaptation is needed. Calls for such guidance have been made elsewhere [48,49] and this study underscores this need.

As noted above, a large proportion of the SHE research has focused on exploring the impact of comprehensive curricula on health outcomes. In part, this is due to the United States’ history of controversy and cultural constraints surrounding SHE, resulting in tensions within communities regarding what kind of programming to implement [50,51,52]. The evidence base for comprehensive SHE therefore has been used to defend the use of comprehensive programs as opposed to the adoption of abstinence-focused programs. 

This “defensive” approach has resulted in the exclusion of deeply exploring other models of education [53]. There have been calls to move beyond merely advocating for “comprehensive” sex education that focus on STI prevention and reproductive health, and move towards a more holistic SHE that “encompasses physical, emotional, mental and social well-being in relation to sexuality” [54] (p. 22) and is explicitly inclusive and intersectional [55,56]. Some have called for specifically anti-oppressive SHE [53]. Anti-oppressive SHE is student driven, creates a safe space for all students, and allows teachers the ability to use their practice-based evidence to ensure their delivery is attuned to student needs. Such an approach aligns strongly with what students and teachers in this study recommended and with the tenets of critical pedagogy. Rather than advocate for a different approach, others have advocated for adapting the definition of comprehensive SHE to be more inclusive of these topics and practices [57]. In either case, critical pedagogy provides a useful framework for understanding how to push SHE closer to what advocates, young people, and educators say is needed.

Indeed, examples of such curricula, largely developed by BIPOC and LGBTQ+ educators, do exist. In fact, one example (a curriculum on communication skills and healthy relationships by the Women of Color Sexual Health Network–Communication Mixtape: Speak On It! Volume 1 [58]) addresses all four recommendations made in this study. This curriculum includes lessons on sexuality messages, types of propaganda, how the media portrays Black bodies, and sexual pleasure—which address this study’s third and fourth recommendations. Further, these lessons include guidance on how lessons may be adapted based on needs of the students, including guidance on assessing the accessibility of different activities for students with diverse learning needs and recommendations for the use of more discussion-based activities or experiential activities based on the size of the group and the relationships of students within the group have with one another. These teaching strategies address this study’s first and second recommendations.

Further, the fact that such curricula are being developed by BIPOC educators mirrors the fact that in this study the only teachers who explicitly spoke about addressing inequity and racial identity were Black teachers. In addition to the need to increase the proportions of teachers of color that many educators and advocates have called for [59], there is a need to ensure curricula, including SHE curricula, are designed and informed by BIPOC educators. There is a need to focus training and technical assistance on the development of skills in discussing race and identity in SHE classrooms. The use of a critical pedagogy framework in SHE would help to center this important aspect of preparation or sexual health educators.

### Limitations

The sample size in this study is appropriate for the exploratory nature of this qualitative study. There were 16 interviews in this study, which exceeds the recommended 12 needed to ensure saturation [60]. The use of five focus groups aligns with recommendations for achieving focus group saturation [61]. However, selection bias is a likely limitation. The teachers who chose to be enrolled in this study likely represent a group that is generally more open to discussing and critiquing SHE. Further, the fact that three of the five student focus groups were recruited through GSAs, which tend to focus on social justice issues and activities, may indicate that this sample is more likely than the larger 9th-grade CPS student population to acknowledge the importance of intersecting racial, ethnic, and LGBTQ+ identities as well as other topics related to LGBTQ+ inclusivity.

Additionally, the timing of this study may be important in interpreting these results. Had data been collected more recently, following the murder of George Floyd and the most recent wave of protests and racial reckoning in the United States, White teachers’ consciousness and awareness of the need to speak more explicitly about race and racism in the classroom may have shifted, and whereas White teachers did not speak explicitly about the need to center race in discussions of sexual health inequities, it is possible they may speak more explicitly about it now.

Member-checking conversations with key stakeholders, including students, helped to ensure trustworthiness of findings. However, although the lead researcher engaged in reflection and peer-debriefing activities to be more conscious of her own biases, age and White racial identity may have influenced how she perceived the urban, mostly non-White students during data collection and analysis.

## 5. Conclusions

Although neither teachers nor students named it as such, the recommendations made by students and teachers in this study align with a critical pedagogical approach. As Darder, a student of Freire and critical pedagogue, has emphasized, the process of meaningful education is a process that requires collective action on the part of teachers, students, their colleagues, and the larger school community. Darder states that this process is essential to develop a classroom pedagogy that “serves students’ context specific needs” [62] (p. 350). Ladson-Billings [24] suggests that if teachers can investigate what is going on in the lives of their students, then they can shape their pedagogy and curriculum to better reflect these lives. It follows then that SHE be no exception and that teachers be empowered to use curricula as a tool to better ensure student learning and that students experience SHE that is asset based, attuned to their lived experiences, and challenges the status quo.

## Figures and Tables

**Table 1:** Types of schools included in this study, *n* = 16.

School Characteristics	Number	Percent
**School type**		
Charter	1	6.0
City-wide option	1	6.0
Magnet	1	6.0
Military academy	1	6.0
Neighborhood	7	44.0
Selective enrollment	5	31.0
**School demographic profile**		
Majority African American/Black	5	31.0
Majority Hispanic/Lantinx	5	31.0
Majority African American/Black or Hispanic/Latinx	2	12.0
Mix of African American/Black, Hispanic/Latinx, White, and Asian students	4	26.0

**Table 2:** Teacher and student demographic characteristics.

Characteristic	Teachers, *n* = 16		Students, *n* = 46	
	Number	Percent	Number	Percent
**Gender**				
Female/Woman/Girl	8	50.0	27	59.0
Male/Man/Boy	8	50.0	16	35.0
Non-binary	0	0	2	4.0
Not reported	0	0	1	2.0
**Age**				
14–17	-	-	46	100%
25–34	8	50.0	-	-
35–44	5	31.0	-	-
45–54	3	19.0	-	-
**Race/ethnicity**				
Black or African American	3	19.0	16	35.0
Latino/Hispanic	0	0	26	57.0
White or European American	13	81.0	2	4.0
Bi-/Multi-racial	0	0	2	4.0
**Teacher type**				
Health/PE Teacher	15	94.0	-	-
Special Education Teacher	1	6.0	-	-

**Table 3:** Key themes from this study, example quotations supporting key themes, and alignment with critical pedagogy concepts.

Key Themes in This Study	Example Quotations Supporting Key Themes	Alignment with Characteristics of Critical Pedagogy
Incorporate more student-centered learning opportunities and allow for tailoring to each specific group of students	*Do I start off the class in a circle, because I know that this needs to be a very structured, formal conversation? Or if I know that my class is a little more mature and they can handle a conversation like that when you’re talking about things such as sexual assault, can it be more of an open format where kids are allowed to freely speak however they want? It really just depends on the student body itself, and it changes from year to year.* Teacher #4	Develop a classroom pedagogy that centers students; and Do not revert to the “banking model” of education, in which students are viewed as passive receptacles of information [33]. Prioritize the voices and lived experiences of the students in their classrooms [20].
2. Use discussion and dialogue to encourage students’ exploration of their own opinions and identities and development of a sense of agency over their own learning	*I asked her if we could do a fishbowl so that we could talk more that [about different opinions on when it’s ok to have sex], cause I like fishbowl talks… everybody writes a question and you don’t have to put your name on it and put it in the bowl and be pulled... Because I feel like sex ed shouldn’t really be a lesson. It should be more of a discussion, an open discussion.* Focus group #3	Create conditions for students to develop a sense of agency for their learning [23,24]. Consider students active “executors” of the learning process [22].
3. Shift focus from risk reduction to a more holistic focus on healthy sexual wellbeing	*I feel like just talking about how all the different ways like people could have sex because they didn’t talk about sex is for pleasure at all. …They mentioned sex for reproduction, that’s it.* Focus group #3	Use an asset-based orientation, focusing on strengths, resources, and resilience; and Move away from deficit approaches to address “lacks” such as poverty and unemployment [34].
4. Directly discuss current health inequities, contributing factors, and intersectionality	*If I was going to make my curriculum about this, make sure to not make it cis or heteronormative… So when they try to bring this and point out to somebody who’s non-conforming or trans, they’re like, “There’s only two genders, there’s only two sexes.” You are absolutely wrong… This is ‘Wham!’ Those myths can be shattered… Just talk about that make sure they acknowledge their privilege when they talk about certain things and make sure to acknowledge the question at the same time… There are definitely nuances. I feel like it’s damaging. It can be harmful [not to address those topics].* Focus group 5	Encourage students to question, critique, and contest structural inequities, norms, and ideas embedded therein [33,35].
